# Towards “greener” strategies in quality control: rapid volatilomics of cocoa based on HS-GC-IMS and machine learning

**DOI:** 10.1007/s00216-026-06415-3

**Published:** 2026-03-10

**Authors:** Lukas Bodenbender, Sascha Rohn, Hadi Parastar, Katrin Sinderhauf-Gacioch, Philipp Weller

**Affiliations:** 1 Institute for Instrumental Analysis and Bioanalytics, Technische Hochschule Mannheim, Paul-Wittsack-Str. 10, 68163 Mannheim, Germany; 2 Department of Food Chemistry and Analysis, Institute of Food, Technology and Food Chemistry, Technische Universität Berlin, KAA 1-2, Kaiserin-Augusta-Allee 14, 10553 Berlin, Germany; 3 Department of Chemistry, Sharif University of Technology, P.O. Box 11155-9516, Tehran, Iran; 4 Alfred Ritter GmbH & Co. KG, Alfred-Ritter-Strasse 25, 7111 Waldenbuch, Germany

**Keywords:** HS-GC-IMS, VOC profiling, Cocoa liquor, Food authentication, Chemometrics, Green analytical chemistry

## Abstract

Gas chromatography-ion mobility spectrometry (GC-IMS) has emerged as a powerful analytical platform in quality control of food, beverages, and flavor products. The technology allows for point-of-care application without the need for sample preparation, which makes it advantageous in resource-limited and equipment-hostile environments. One of these fields is the quality assessment of raw cocoa, which is fundamental for ensuring authenticity, product quality, as well as food safety and compliance. At the same time, analytical departments are facing an increasing urge to turn to a more sustainable use of resources, as well as substantial cost pressure. In the present study, a fast GC-IMS strategy was used to evaluate the provenance of cocoa in combination with machine learning. While most of the commercially available GC-IMS systems are based on nitrogen as a carrier gas, this approach was optimized and translated to a fast, hydrogen-based GC method. This was applied to a set of commercial cocoa liquor and data were evaluated by machine learning approaches, such as multivariate curve resolution-alternating least squares (MCR-ALS) and partial least squares-discriminant analysis (PLS-DA). By cutting down the analysis time by a factor of 2.5, this study demonstrates that in contrast to most conventional gas chromatography-mass spectrometry (GC-MS) systems, GC-IMS can be easily optimized towards higher throughput using the faster flow rates possible with hydrogen. Furthermore, this leads to enhanced signal quality and thus, a better basis for machine learning and finally, to an optimal tool for the classification of raw cocoa origins. Therefore, H_2_-based GC-IMS can be considered as a greener, resource-friendly, and efficient approach for the analysis of volatile food and beverage samples.

**Graphical abstract:**

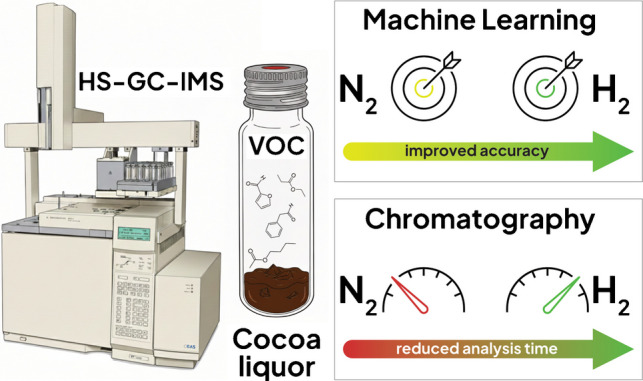

**Supplementary Information:**

The online version contains supplementary material available at 10.1007/s00216-026-06415-3.

## Introduction

Green analytical chemistry is gaining increasing interest in the fields of quality control and food authenticity, particularly driven by the need for environmentally sustainable, cost-efficient, and high-throughput analytical methods. One of the main drivers of costs and use of resources is the commonly required sample preparation. In this context, the term “volatilomics” has evolved as a synonym for omics-based, gas-phase analysis of complex food, beverage, and flavoring samples and has gained more and more relevance within the last years. The combination of non-targeted analytical strategies and advanced data analysis techniques provides comprehensive sample insights and offers a high potential for automation and subsequently a more robust data analysis [1, 2]. Non-target screening (NTS) detects chemical features beyond conventional target or suspect analyses and enables the acquisition of extensive profiles from complex samples, resulting in multidimensional fingerprints that enhance identification accuracy [3]. Consequently, the integration of efficient processing of NTS approaches and further chemometric methods is essential for the interpretation of complex chromatographic datasets. Modern data analysis strategies, relatively basic ones, such as principal component analysis (PCA), partial least squares (PLS), multivariate curve resolution (MCR), or more advanced ones, such as ensemble learning (EL) and artificial neural networks (ANNs), facilitate both qualitative and quantitative analysis by reducing data dimensionality and enhancing pattern recognition [1].

Due to their simple and robust hardware, the small footprint and the operation principle, GC-IMS systems have found their way into food authentication strategies in various fields and applications [4–8]. While nearly all food materials have quality issues or are subject to adulteration such as obvious for olive oil, honey, and coffee, cocoa is also one of the raw materials that showed substantial price increases over the last three years. As such, the authenticity and quality control of cocoa (*Theobroma cocoa* L.) is an increasingly important issue, in particular with regard to provenance, off-flavors, and traceability for consumers and industry. The composition of cocoa liquor from different geographic origins varies, for instance, due to different cocoa varieties (e.g., *Criollo*, *Forastero*, *Trinitario*, *Nacional*), fermentation, and roasting as well as technical processes [9–11]. With regard to flavor and aroma, fermentation and roasting are crucial steps with a distinctive influence on the final product [12–15]. Cocoa liquors feature a broad spectrum of VOC, including ketones, aldehydes, terpenes, pyrazines, alcohols, and esters, which differ distinctly based on the cultivar, geography, and growing conditions [12, 16, 17]. While West African liquors are typically of the “bulk” variety *Forastero* and are characterized by a rather “standard” cocoa flavor with nutty undertones, South American liquors tend to display more floral and fruity notes [10, 18]. However, the South American fine flavor cocoas of the varieties *Criollo*, *Trinitario,* and *Nacional* cover only approximately 5–10% of the cocoa world production [10, 19]. In the field of quality analysis of cocoa beans, liquors, and nibs, GC-MS is a widely used technique. There are numerous approaches for the analysis of the volatile fingerprint of cocoa samples, mostly using SPME-GC-MS, which focus on the differentiation of roasted and unroasted cocoa beans, the stage of fermentation and drying processes, cocoa provenance, or the key VOC of roasted beans, liquor, and chocolate [12, 14, 17, 19]. In combination with chemometric analysis, HS-SPME-GC-MS has been described by several studies to assess the origin of cocoa beans and liquors, demonstrating potential for non-targeted fingerprinting. These approaches enable effective discrimination of cocoa origin, with targeted VOC profiling offering additional insight into key odorants and biochemical pathways linked to processing conditions. Nonetheless, as described, e.g., by Bagnulo et al*.*, some rather fruity, potentially characteristic compounds for South American cocoa liquors remain inconsistent in the literature, while compounds with a nutty odor description, such as pyrazines, are present in higher concentrations in West African cocoa samples [17–19]. A further study describes non-targeted studies for the analysis of the VOC profile of alkalized and natural cocoa using stir bar sorptive extraction (SBSE) in combination with GC-MS and chemometric data evaluation [13]. Most of the GC-MS-based studies describe around approx. 60 VOC, having in common a rather lengthy sample extraction as well as comparatively long GC methods of approx. 40 min. or more. One reason for these time-consuming GC methods is that most of the GC-MS systems are operated using helium, which still is the standard carrier gas for GC-MS systems. This is however limiting the carrier gas velocity distinctively in comparison to hydrogen. Typical linear velocity in routine environments ranges around 40 cm/s for helium, while hydrogen allows flows of 70 cm/s and more without a substantial loss of separation quality. As stated before, it is feasible to operate GC-MS systems with hydrogen; however, the resulting EI spectra are often deviating from standard databases, such as NIST, which often pushes users back to helium. In contrast, GC-IMS is highly flexible in the choice of carrier gases and excels when hydrogen is used.

As typical GC analysis times for complex food samples range up to 60 min., there is an increasing interest in faster chromatographic separations. While faster GC methods mean higher sample throughput and, as such, more efficient use of resources, it remains essential to maintain resolution, sensitivity, and peak shape [20]. There are a number of options to speed up chromatographic methods, such as faster oven temperature ramp programs, reduced internal column diameters and shorter column length, lower film thickness, and the choice of stationary phases, but one of the simplest and most efficient ones is the use of hydrogen as the GC carrier gas and an increased flow velocity [20, 21]. Thus, it is possible to decrease the chromatographic separation times by a factor of 3 to 10 without a severe loss of resolution if all parameters are optimized. The use of hydrogen as carrier gas as a sole parameter already is a particularly straightforward and impactful enhancement, typically doubling the separation speed relative to helium and even more regarding nitrogen [21]. A further reason is that helium usually is obtained from natural gas resources and is substantially more expensive than hydrogen or nitrogen. The latter is theoretically feasible as a carrier gas for GC-MS, but does not have any practical relevance due to the high ion background. In contrast, GC-IMS typically is based on nitrogen as the carrier gas of choice, mainly because these systems are operated under atmospheric conditions and therefore are nearly indifferent to gas viscosity [22–24]. Although initial studies have explored the use of hyper-fast GC systems, employing hydrogen as a carrier gas in combination with DTIMS, such systems are still in progress and currently associated with relatively high buying costs, which limits their use in routine environments [25]. Still, hydrogen is superior as a carrier gas in comparison to nitrogen and helium when it comes to the optimal height of theoretical plates (HETP) versus higher linear velocities, as given by the van Deemter equation [21]. Hydrogen is often considered too hazardous to be used as a carrier gas; however, modern H_2_ benchtop generators provide an easy access to safe and high-purity hydrogen without the need for additional gas cylinders anymore.

For GC-hyphenated IMS setups, drift tube IMS systems (DTIMS) are the most widely applied configuration. Further approaches are based on aspiration ion mobility (AIMS), differential ion mobility (DMS or DIMS), or field asymmetric waveform ion mobility (FAIMS) [26–28]. For the sake of simplicity, the term IMS is used in the context of DTIMS. In IMS, ions are accelerated against a defined drift gas in a drift region. Each analyte features a certain drift time due to collisions with drift gas molecules. This is dependent on its geometric structure, mass, charge, and further on temperature, the drift gas, and the electric field strength [29, 30]. The ionization is a reaction cascade initiated by collisions of electrons with the drift gas atmosphere. Depending on the moisture and temperature of the drift gas atmosphere, proton-water clusters, H^+^[H_2_O]_n_, are formed, also termed reactant ions [29, 31, 32]. With an adduct ion formation, due to collisions between sample molecules and reactant ions, protonated monomers, MH^+^[H_2_O]_n−x_, and with a high analyte load in addition protonated dimers, M_2_H^+^[H_2_O]_n−x_, are formed [29, 33, 34]. In contrast to traditional approaches, such as GC-MS, which typically require an online enrichment step, such as solid-phase micro extraction (SPME) or in-tube extraction (ITEX), this is usually not required in headspace-based GC-ion mobility spectrometry (GC-IMS) due to the higher sensitivity for polar and medium-polar substances as compared to fullscan quadrupole MS [23, 35]. Therefore, the integration of HS-GC-IMS with chemometric workflows enhances analytical throughput while reducing operational demands, including energy consumption, solvent use, and waste generation. Consequently, GC-IMS demonstrates superior sustainability and analytical greenness compared to HS-GC-MS and SPME-HS-GC-MS, primarily due to reduced sample preparation, the capability of faster chromatographic separations, and the absence of vacuum systems, as recently reported by Parastar et al*.* [23]. In this context, GC-IMS offers itself as a feasible option for a faster and greener strategy for quality and authenticity analysis of cocoa raw materials; however, surprisingly, the application of GC-IMS to the analysis of cocoa nibs, liquor, or chocolate described in the literature to date is scarce. While there are studies for the monitoring of VOC during the chocolate production and allow for discrimination of different production processes and products such as cocoa liquor, nibs, or chocolate using PCA, there are to date no applicative methods for routine analytics of cocoa samples, and existing methods often lack sufficient sensitivity in sampling and compound identification [36, 37].

The aim of the present study was to evaluate the applicability of HS-GC-IMS with hydrogen as a carrier gas for the analysis of VOC in comparison to the commonly used carrier gas nitrogen. Existing analytical approaches in the field of cocoa authenticity and quality control primarily are based on the use of HS-SPME-GC-MS with comparatively long analysis times, and the application of hydrogen as a carrier gas for commercially available HS-GC-IMS is to date not described in literature. In this work, a dataset of 60 cocoa liquors with four different provenances was used to investigate the applicability of a fast HS-GC-IMS method using hydrogen as a carrier gas and the subsequent influence on classification performance of machine learning algorithms.

## Materials and methods

### Reagents and samples

The dataset included 60 commercial cocoa liquors, representing four countries of origin: Ghana (9), Ivory Coast (28), Nicaragua (6), and Peru (17). Cocoa liquors were produced from locally fermented and dried at origin cocoa beans in commercial cocoa factories in Europe, using the process steps of roasting (bean roasting or nibs roasting), breaking, winnowing, and grinding (knife mill and ball mills for fine grinding). All samples were stored tightly sealed at −20 °C under exclusion of light. Prior to the measurements, the cocoa liquors were heated gently in a water bath at 50 °C, and after melting, 2.5 g of each liquor was transferred to a 20 mL headspace vial and sealed with a screw cap. All samples were prepared and analyzed in duplicates.

For substance identification, several analytical standards were used. All reference compounds were purchased either from Sigma-Aldrich Chemie GmbH (Taufkirchen, Germany), Alfa Aesar by Thermo Fisher GmbH (Kandel, Germany), or Carl Roth GmbH & Co. KG (Karlsruhe, Germany) at the highest available purity. The HS-GC-IMS instrumentation and parameters were identical to the cocoa samples. The reference chemicals were either dissolved in water or in fresh sunflower oil, depending on their solubility. Tested compounds were 2,3-butadione, ethyl acetate, 2-methyl-1-propanol, 3-methylbutanal, 3-methyl-2-butanol, ethyl propanoate, 3-hydroxybutan-2-one, 3-methyl-1-butanol, ethyl isobutyrate, 2,3-butanediol, butyl acetate, furfural, isoamyl acetate, β-myrcene, ethyl hexanoate, hexyl acetate, limonene, phenylacetaldehyde, linalool oxide, linalool, nonanal, 2-phenylethanol, and ethyl octanoate. Additionally, a ketone standard (C_4_-C_9_), containing 2-butanone, 2-pentanone, 2-hexanone, 2-heptanone, 2-octanone, and 2-nonanone, was used for system normalization of the retention times (RT) and subsequent library-based substance identification, using retention indices (RI) and compound-specific drift times (DT). In GC-IMS, signals can be identified by the combination of retention and drift time. While retention indices in GC-IMS often correspond to multiple compounds, the incorporation of IMS drift times significantly enhances specificity, as the combined RI and DT values typically provide a unique signature for each compound. For library search, the software VOCal 4.12 was used (Gesellschaft für analytische Sensorsysteme mbH, Dortmund, Germany).

### Instrumentation

Measurements were performed with a Focus-HT-IMS system (Gesellschaft für analytische Sensorsysteme mbH, Dortmund, Germany) coupled to a gas chromatograph 6890 N (Agilent, Santa Clara, CA, USA), equipped with a CombiPAL headspace sampling unit (CTC Analytics AG, Zwingen, Switzerland) as, e.g., described in previously published studies by our group [38]. The incubation temperature was set to 100 °C for 12 min. with 400 rpm. A headspace volume of 1 mL was injected using a gas-tight 2.5 mL syringe (Trajan Scientific and Medical, Ringwood, Australia). To avoid condensational and cross contamination effects, the headspace syringe was set to 105 °C and the syringe was flushed for 5 min. after each injection with nitrogen. On an HP-5 capillary column (operating temperature: − 60 °C to 325 °C/350 °C; SN: USB345942H, Agilent Technologies, Santa Clara, CA, USA) chromatographic separation was performed with dimensions of 30 m × 0.32 mm × 0.25 μm film thickness. Injection was performed using a split/splitless injector at 200 °C and a split ratio of 1:20. The measurements were accomplished twice, with nitrogen and hydrogen as a carrier gas, respectively. The measurements with nitrogen, purity 99.99%, were carried out at constant inlet pressure of 6.7 psi resulting in an initial velocity of 26.3 cm/s. GC oven was programmed from 40 °C, holding two min., to 120 °C with 5 °C/min and from 120 °C to 200 °C with 10 °C/min resulting in a runtime of 26 min. For the use of hydrogen, an electrolysis gas generator (HG PRO 260, LNI Swissgas GmbH, Kamen, Germany) was used. The resulting purity of the electrolysis hydrogen is > 99.99999% and the GC inlet pressure was set to constant 8.95 psi with an initial velocity of 70.0 cm/s. The oven ramp was programmed with an initial time of 1 min. at 40 °C and afterwards a ramp of 15 °C/min to 120 °C and 30 °C/min to 200 °C, holding for 1 min., resulting in an overall runtime of 10 min. The transfer line to the IMS was set to 200 °C for all measurements and the IMS cell was heated to 120 °C. The Focus-HT-IMS setup is built with an ^3^H ionization source (approx. 100 MBq ß-emission). The drift tube of the setup had a diameter of 15.2 mm and a length of 53 mm, consisting of stainless steel and PEEK. For the measurements, IMS was operated in positive ion mode at a constant voltage of 2.5 kV. The injection voltage was set to 2500 a.u. and the blocking voltage to 70 a.u., respectively, which are software settings in arbitrary units. Drift gas flow was controlled using a mass flow controller at 150 ml/min (Voegtlin Instruments AG, Aesch, Switzerland). For noise and file size reduction, each recorded spectrum was averaged of six scans using a repetition rate of 21 ms, an injection pulse width of 100 μs, and a sampling frequency of 228 kHz.

### Data processing and evaluation

The spectra of both datasets were evaluated using *gc-ims-tools* 0.1.7 [39]. To reduce excessive RAM usage due to the large number of variables in the spectra, both datasets were subjected to a “db3” wavelet compression with a level of 2. In addition, the datasets were aligned in the direction of drift time to avoid shifts in the spectra. As the drift time is dependent on the atmospheric pressure, this is a crucial preprocessing step. Further drift times were normalized to the RIP. RIP position therefore is accessed by searching the intensity matrix for the drift time of the maximum value. For the dataset using nitrogen as a carrier gas, the spectra were cut at 100 s and 1500 s, and the spectra using hydrogen were cut in a range of 50 s and 600 s, whereas for both datasets the range of 1.03–2.5 (approx. 3.8–9.5 ms) in the RIP relative drift time axis were kept.

After preprocessing, both datasets were split into 80% training and 20% validation data. For partial least squares-discriminant analysis (PLS-DA), the number of latent variables (LVs) was selected by the minimum root-mean square error of cross-validation (RMSECV), resulting in seven LVs for the dataset of hydrogen and six LVs for the dataset of nitrogen (see Figure S1). Afterwards, the validation data was used to check the model in terms of overfitting and performance. Further, accuracy, sensitivity, and specificity were calculated for the calibration, 5-fold cross-validation, and prediction of the validation data. The accuracy of a model is defined as the ratio of correctly classified samples to all of the samples (see Eq. 1) [40].

1 $$\mathrm{Accuracy}=\frac{\text{Number of correct predictions}}{\text{Number of all predictions}}$$

The sensitivity of each class *k* is also known as recall; it is the ratio of true positives to all positives in the dataset (see Eq. 2), while the specificity describes the ratio of true negatives to all negatives in the data (see Eq. 3) [40, 41].

2 $${\mathrm{Sensitivity}}_{k} =\frac{{\text{True positive}}_{\mathrm{k}}}{{\text{True positive}}_{\mathrm{k}}+{\text{False negative}}_{\mathrm{k}}}$$

3 $${\mathrm{Specificity}}_{k} =\frac{{\text{True negative}}_{\mathrm{k}}}{{\text{True negative}}_{\mathrm{k}}+{\text{False positive}}_{\mathrm{k}}}$$

Classification was applied to the dataset of cocoa with different geographical provenance, and the metrics were evaluated for both datasets, with the carrier gases nitrogen and hydrogen, respectively. Besides the 5-fold cross-validation, a leave-one-out cross-validation (LOO-CV) and venetian blind cross-validation were applied on the dataset to evaluate the model performance. In stratified *k*-fold cross-validation, the dataset is randomly partitioned into *k* disjoint folds of approximately equal size, maintaining the original dataset’s class distribution in each fold. Afterwards, each fold is then used once as a test set, while the remaining *k *− 1 folds are used to train the model [42]. In venetian blind cross-validation (VB-CV), each test set is created by selecting every *i*th object in the dataset, starting at objects numbered 1 through *i* [40]. In LOO-CV, each object in the dataset is omitted from the dataset and used as a test set, while the analysis is performed on the remaining data. This process is repeated for every observation, enabling the model to be validated on multiple test sets [40, 43].

For MCR-ALS, the GC-IMS data for different samples were first column-wise augmented with retention times of different samples as rows and drift times as columns. This augmented data was then resolved using MCR-ALS. First, singular values obtained from singular value decomposition (SVD) were used for estimation of the number of components in each dataset. Then, *simple-to-use interactive self-modelling mixture analysis* (SIMPLISMA) was used to calculate the initial estimates of spectral profiles to start ALS optimization. Additionally, different constraints were applied during MCR-ALS including spectral normalization, non-negativity in both GC and IMS modes, unimodality in GC mode, and component correspondence. Lack of fit (LOF) was used as a metric to evaluate the performance of the MCR-ALS algorithm. The MCR-ALS results provided pure GC and IMS profiles for 60 metabolites, with the IMS profiles allowing chemical identification. In parallel, the MCR-ALS-enhanced GC profiles were refolded, and peak areas were calculated for each resolved metabolite across all samples. This procedure produced a peak table consisting of 60 cocoa samples (rows) and 60 metabolites (columns), which was subsequently subjected to multivariate analysis using PLS-DA. MCR-ALS calculation was performed using MCRALS toolbox version 2.0 in MATLAB R2019b (The MathWorks, Natick, MA, USA).

## Results and discussion

**Fig. 1:**
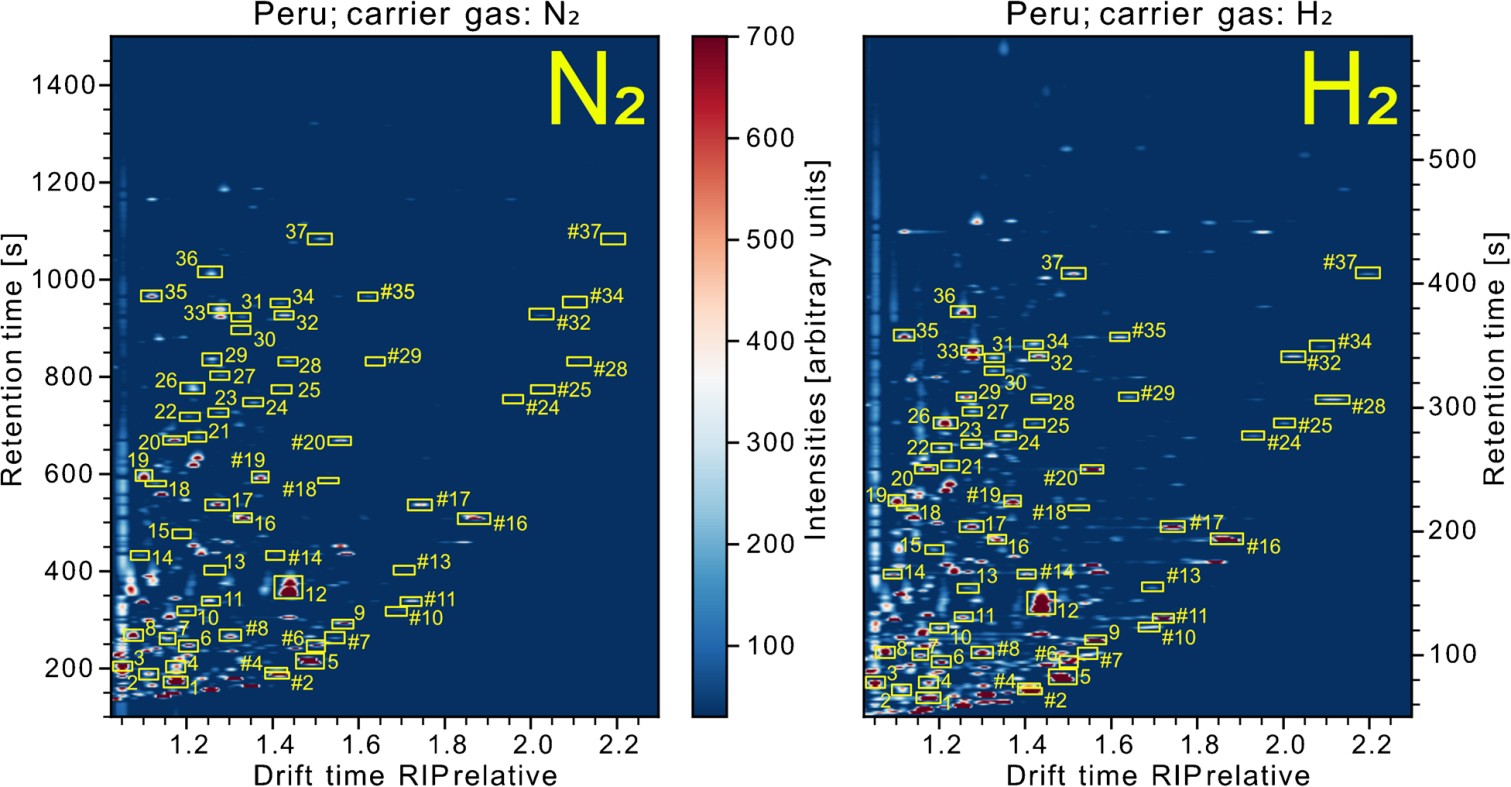
Exemplary GC-IMS spectra of a cocoa liquor with nitrogen (left) and hydrogen as carrier gas (right), respectively. The annotated substances are listed in Table 1. Dimers are marked with a #

In Fig. 1, an HS-GC-IMS spectrum of raw cocoa obtained with the carrier gas nitrogen and one with the carrier gas hydrogen is depicted, respectively. Peaks, identified through analytical standards or library searches, are annotated in Fig. 1 and summarized in Table 1. The corresponding dimer peaks are denoted with # in the figure. Due to the monomer and dimer peaks of the reference analytical standards, the identification of substances by the combination of retention and drift times is possible. All of the annotated and identified volatile compounds were described in previous research and range from alcohols, ketones, and aldehydes to esters, terpenes, pyrazines, and furans [13, 14, 17–19, 44]. The experimental retention indices of the identified compounds, obtained with hydrogen as the carrier gas, are in accordance with existing literature and show a difference of maximum ± 15, while most of them are in a range of ± 5 [12, 45–51].

Although more than 30 compounds were identified in the present study, a further refinement of GC-IMS databases is required to improve compound annotation. By comparison, enrichment-based approaches such as SPME-HS-GC-MS have previously yielded nearly twice as many identified constituents [17, 19]. Nevertheless, it should be noted that no enrichment techniques were employed in this study, and the differences in sensitivity are not substantial, as several compounds remain unannotated. This underlines the advantages of HS-GC-IMS, which offers superior sensitivity for polar and medium-polar VOCs and enables higher sample throughput due to the absence of additional sample preparation steps, making it a powerful tool for food and flavor analysis [23].

**Table 1:** List of identified substances in the cocoa liquors. The columns show the annotated number, the substance, retention index in literature and experiment, as well as the identification by an analytical standard (STD) or the library of retention time x drift time library (RI x IMS)

Annotated number	Compound	CAS number	RI^exp^	RI^literature^	Identification
1	Butane-2,3-dione	431-03-8	593	586 [50], 607 [47]	STD, RI x IMS
2	Ethyl acetate	141-78-6	609	612 [45], 613[46]	STD, RI x IMS
3	Acetic acid	64-19-7	610*	591 [45], 658 [47]	RI x IMS
4	2-Methylpropan-1-ol	78–83-1	616	622 [45], 619 [46]	STD, RI x IMS
5	3-Methylbutanal	590-86-3	640	644 [47], 648 [50], 649 [45]	STD, RI x IMS
6	3-Methylbutan-2-ol	598-75-4	692	692 [52]	STD, RI x IMS
7	Ethyl propanoate	105-37-3	700	709 [46]	STD, RI x IMS
8	3-Hydroxybutan-2-one	513-86-0	705	711 [45, 46]	STD, RI x IMS
9	3-Methylbutan-1-ol	123-51-3	731	730 [50], 737 [46], 738 [45]	STD, RI x IMS
10	Ethyl 2-methylpropanoate	97-62-1	746	746 [49], 768 [12]	STD, RI x IMS
11	2-Methylpropyl acetate	110-19-0	767	753 [46], 759 [12]	STD
12	Butane-2,3-diol	513-85-9	775	782 [46]	STD
13	Butyl acetate	123-86-4	808	812 [51], 816 [46]	STD
14	Furfural	98-01-1	826	825 [12], 830 [50], 836 [51]	STD, RI x IMS
15	2-Methylbutanoic acid	116-53-0	861	851 [45], 859 [49]	RI x IMS
16	3-Methylbutyl acetate	123-92-2	876	876 [51], 878 [49], 880 [46]	STD, RI x IMS
17	Heptan-2-one	110-43-0	894	873 [12], 890 [50]	STD, RI x IMS
18	2,6-Dimethylpyrazine	108-50-9	907	915 [45], 920 [47]	RI x IMS
19	2-Ethylpyrazine	13925-00-3	920	910 [47], 915 [50]	RI x IMS
20	Benzaldehyde	100-52-7	964	956 [12], 958 [50], 962 [46]	RI x IMS
21	5-Methylfurfural	620-02-0	965	956 [12], 961 [48], 971 [47]	RI x IMS
22	2-Pentylfuran	3777-69-3	990	984 [12], 991 [50], 995 [45]	RI x IMS
23	β-Myrcene	123-35-3	994	981 [12], 989 [46], 991 [51]	STD
24	Ethyl hexanoate	123-66-0	1004	994 [12], 997 [46],	STD, RI x IMS
25	Hexyl acetate	142-92-7	1016	1008 [46], 1009 [12]	STD, RI x IMS
26	Acetyl pyrazine	22047-25-2	1017	1024 [12]	RI x IMS
27	Limonene	138-86-3	1035	1029 [12, 51], 1039 [45]	STD, RI x IMS
28	3-Methylbutyl butanoate	106-27-4	1052	1054 [51], 1064 [12]	RI x IMS
29	2-Phenylacetaldehyde	122-78-1	1057	1052 [12, 48], 1054 [45]	STD
30	cis-Linalool oxide	5989-33-3	1084	1073 [51], 1082 [12]	STD
31	trans-Linalool oxide	34995-77-2	1100	1092 [51]	STD
32	Nonan-2-one	821-55-6	1104	1094 [45], 1105 [12]	STD, RI x IMS
33	Linalool	78-70-6	1108	1099 [49, 51], 1101 [46], 1117 [12]	STD, RI x IMS
34	Nonanal	124-19-6	1117	1113 [48], 1120 [12]	STD, RI x IMS
35	2-Phenylethan-1-ol	60-12-8	1125	1111 [49], 1118 [47], 1124 [45, 48]	STD
36	2,3-Diethyl-5-methylpyrazine	18138-04-0	1153	1154 [49]	RI x IMS
37	Ethyl octanoate	106-32-1	1202	1195 [46, 51], 1198 [48]	STD, RI x IMS

*Tentative identification due to severe peak tailing

With view to run times, it was possible to speed up the analysis time by a factor of 2.5, mainly due to the superior diffusivity and lower viscosity of hydrogen. In comparison to nitrogen, hydrogen permits higher linear velocities and thereby shortens analysis times without compromising chromatographic resolution. However, the spectrum obtained with hydrogen exhibits a notably higher number of signals as well as increased peak intensities. It is important to underline that the complex ionization chemistry of the ^3^H DTIMS system could be changed by a different carrier gas. So far, adverse effects were not observed when using hydrogen as the carrier gas in the sense of excessive artifact formation. One reason could be the fact that unlike in GC-MS systems, temperatures in the ion source area are significantly lower in GC-IMS systems (< 100 °C) and further, the gas composition in the ion source is dominated by an excess of the buffer gas nitrogen (1–2 mL carrier gas flow vs. 150 mL/min buffer gas flow). In the case of non-targeted approaches, ionization-related artifacts are unlikely to affect data interpretation, provided that ionization remains consistent, as non-target screening in GC-IMS relies primarily on pattern recognition rather than on database-driven identification.

For discrimination of the raw cocoa provenance, PLS-DA was employed. PLS-DA integrates dimensionality reduction with classification by maximizing the covariance between the data matrix and the label matrix, making it particularly well suited for high-dimensional datasets with strong collinearity among variables [53, 54]. The optimal number of LVs was determined by evaluating the RMSE as a function of the number of PLS components (LVs). This resulted in seven components for the dataset acquired with hydrogen and six components for the dataset using nitrogen as the carrier gas (see figure S1).

The classification figures of merit of the PLS-DA models, including the results of calibration, cross-validation, and external prediction, are summarized in Table 2. Overall, non-targeted HS-GC-IMS screening combined with multivariate data analysis demonstrated that discrimination between different cocoa mass origins is feasible with both carrier gases. Calibration accuracy was optimal for all classes in both datasets.

**Table 2:** PLS-DA figures of merit for the calibration data, LOO cross-validation, venetian blind cross-validation, 5-fold cross-validation of the test data and the prediction of the external validation data

	N_2_		H_2_	
Calibration accuracy	1.00		1.00	
	Sensitivity	Specificity	Sensitivity	Specificity
Ghana	1.00	1.00	1.00	1.00
Ivory Coast	1.00	1.00	1.00	1.00
Nicaragua	1.00	1.00	1.00	1.00
Peru	1.00	1.00	1.00	1.00
**LOO-CV accuracy**	0.88		0.96	
**VB-CV accuracy**	0.87		0.94	
**5-fold CV accuracy**	0.88		0.96	
	Sensitivity	Specificity	Sensitivity	Specificity
Ghana	0.90	0.96	0.93	1.00
Ivory Coast	0.84	0.97	1.00	0.93
Nicaragua	0.80	0.98	0.90	1.00
Peru	1.0	0.95	1.00	1.00
**Prediction accuracy**	0.92		1.00	
	Sensitivity	Specificity	Sensitivity	Specificity
Ghana	1.00	0.91	1.00	1.00
Ivory Coast	0.86	1.00	1.00	1.00
Nicaragua	1.00	1.00	1.00	1.00
Peru	1.00	1.00	1.00	1.00

**Fig. 2:**
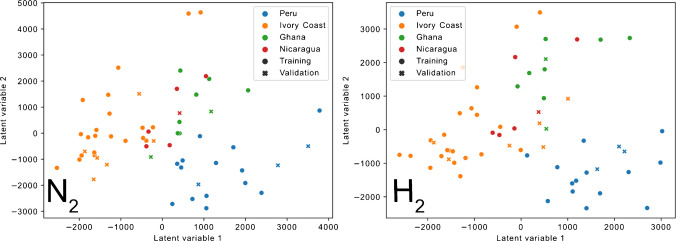
First and second latent variables of the PLS-DA models with the datasets using the carrier gases N_2_ (left) and H_2_ (right) to classify the cocoa masses by provenance, respectively

Different CV strategies confirmed the robustness of the models, with marginally higher accuracies achieved using hydrogen compared to nitrogen. Class-specific metrics revealed particularly high sensitivity and specificity for Peru, whereas slightly lower sensitivities were observed for Ghana, Ivory Coast, and Nicaragua. These figures of merit were again slightly higher in the dataset acquired with hydrogen compared to nitrogen. Furthermore, the external prediction accuracies of 0.92 for nitrogen and 1.00 for hydrogen indicate excellent predictive performance and confirm the absence of model overfitting. The observed improvements in classification performance are likely attributable to the higher efficiency of hydrogen at practical flow rates compared to nitrogen, resulting in enhanced chromatographic resolution. Even though nitrogen exhibits the lowest minimum plate height of common GC carrier gases, its optimal efficiency is confined to a narrow range of linear velocities, and due to the steep slope of its van Deemter curve, chromatographic efficiency decreases sharply even at slightly increased flow rates [20]. Consequently, hydrogen is often favored over nitrogen as a carrier gas in gas chromatography due to its superior chromatographic efficiency. Admittedly, hydrogen is to date not widely used as a carrier gas due to risks of explosion. As capillary gas chromatography operates at low flow rates of only a few milliliters per min., and hydrogen diffuses rapidly, critical incidents are unlikely and would occur only in the event of a severe leak. Thus, these risks can be considered rather low [21]. In Fig. 2, additionally, the scatter plots of the first two LVs derived from the PLS-DA models of both datasets are illustrated, respectively. The validation data, indicated by cross-style markers, corresponds to samples from the external prediction set.

**Fig. 3:**
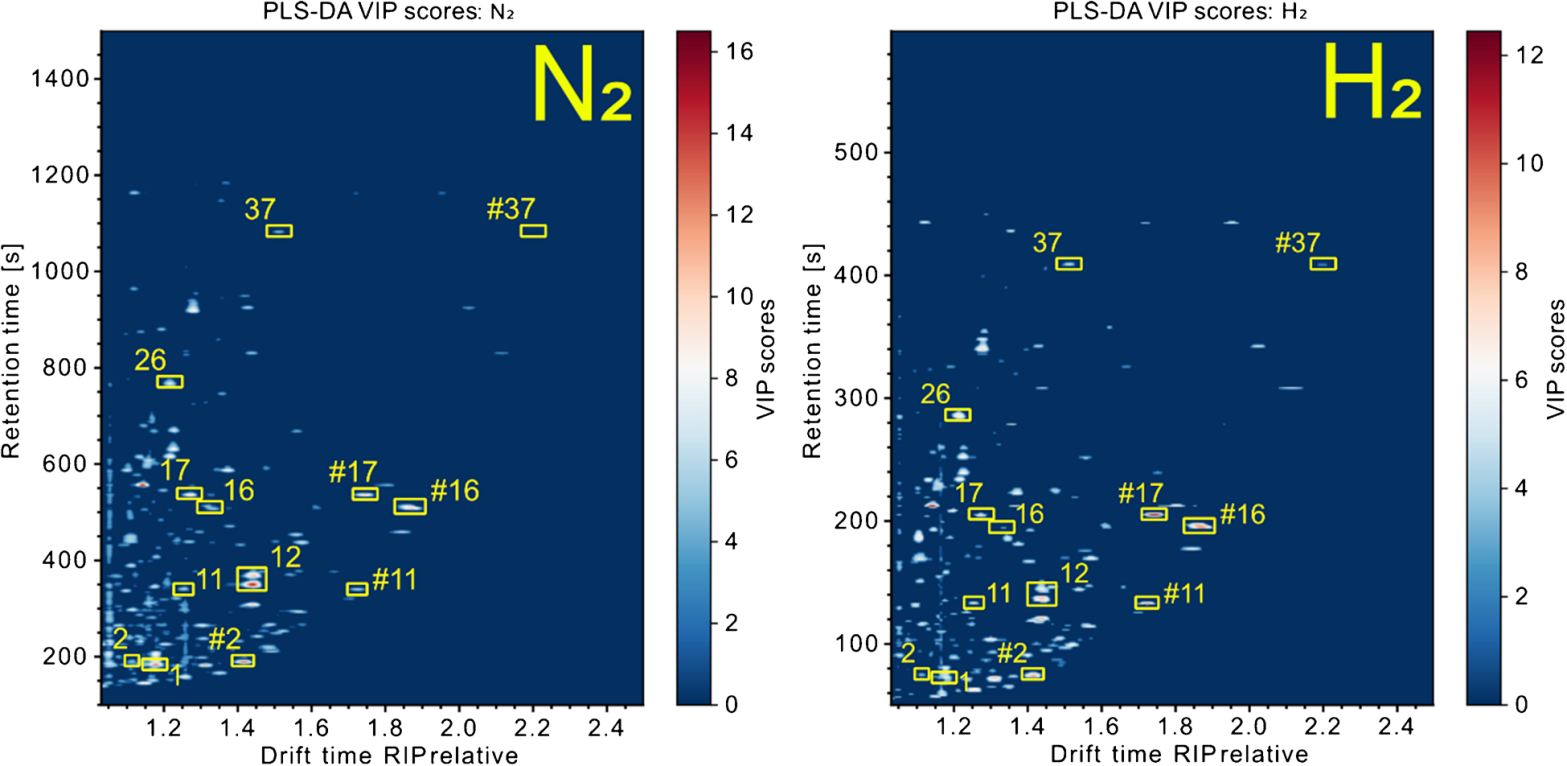
PLS-DA VIP scores of the datasets using the carrier gases N_2_ (left) and H_2_ (right) to classify the cocoa masses by provenance, respectively. The annotated substances are listed in Table 1. Dimers are marked with a #

The first latent variable primarily discriminates in both datasets the cocoa masses from the Ivory Coast from those of other origins, suggesting that this group exhibits the most distinctive VOC profile. The second latent variable separates most of the Peru and Ghana data from the other. Although the Nicaraguan samples exhibit an overlap with other data in this figure, they are effectively discriminated by the other latent variables, as the models perform well for all classes. Another observation is that the axis lengths of the latent variables differ between the datasets, with the latent variables of the hydrogen dataset being more narrowly distributed. Alongside the classification metrics, the PLS coefficients and variable importance in projection (VIP) scores were analyzed for both datasets. Although no characteristic marker compound for any of the cocoa samples was identified, the discrimination among classes can be attributed to relative differences in the ratios between chromatographic peak intensities. This observation is consistent with previous reports in the field of VOC-based analyses of cocoa liquors and beans from different geographical origins [17–19]. The VIP scores reflect a variable’s ability to explain variance in **X** and its correlation with the response. A VIP score above 1 is generally considered significant and VIP scores can be visualized per variable, aiding interpretation and comparison with the original data [55, 56]. In Fig. 3, the PLS-DA VIP scores are visualized with a backwards projection into the original data space.

Due to the backwards projection into the original data space, the previously annotated substances can be marked in the VIP scores plot of the PLS-DA to reveal potentially class-characteristic substances [1]. Differences in feature importance were observed between the carrier gases nitrogen and hydrogen. For clarity, several compounds are highlighted in Fig. 3, with annotations consistent with those listed in Table 1. While the VIP scores of the nitrogen dataset indicate a rather high importance of butane-2,3-dione (1), ethyl acetate (2), and butane-2,3-diol (12), the dataset with the carrier gas hydrogen depicts more and as well different features with a comparatively high importance, such as 2-methylpropyl acetate (11), 3-methylbutyl acetate (16), 2-heptanone (17), or acetyl pyrazine (26). Nonetheless, the higher signal-to-noise level of the hydrogen dataset seems to be beneficial for the model performance and, for instance, the dimer of ethyl octanoate (37) is only represented in the VIP scores of the hydrogen dataset.

**Fig. 4:**
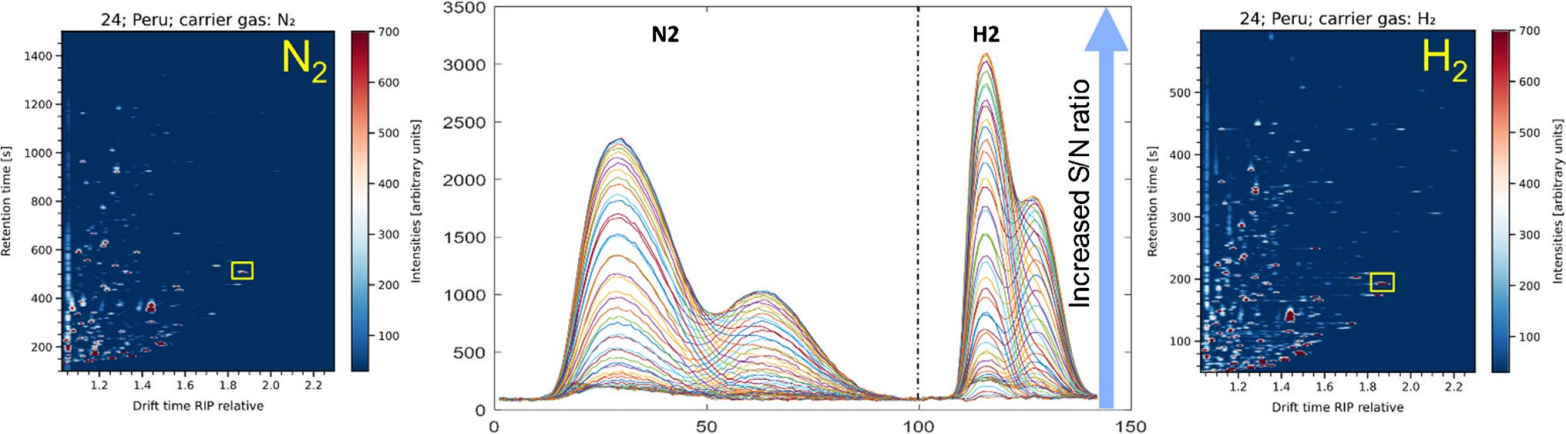
GC peak elution profiles of the highlighted area using the carrier gases N_2_ (left) and H_2_ (right), respectively

To facilitate a more precise evaluation of the peak height and compound separation, a selected region was examined. As shown in Fig. 4, the GC-IMS peak profiles of the highlighted features demonstrate a distinct separation of the two peaks with respect to their GC retention times. Notably, the peak on the right side is substantially narrower, which can be attributed to the use of hydrogen as the carrier gas, and this improved chromatographic efficiency is accompanied by a pronounced increase in peak height.

The first compound is 3-methylbutyl acetate and the second is unidentified yet. Although the two peaks exhibit minimal co-elution in the GC retention time dimension for both datasets, they are still well separated, and their slightly different drift times facilitate data interpretation. Moreover, the peaks obtained with the carrier gas hydrogen depict a higher intensity of roughly a factor of 1.5. This is especially beneficial for small peaks and low abundant analytes, as the signal-to-noise level is affected in a positive way.

**Fig. 5:**
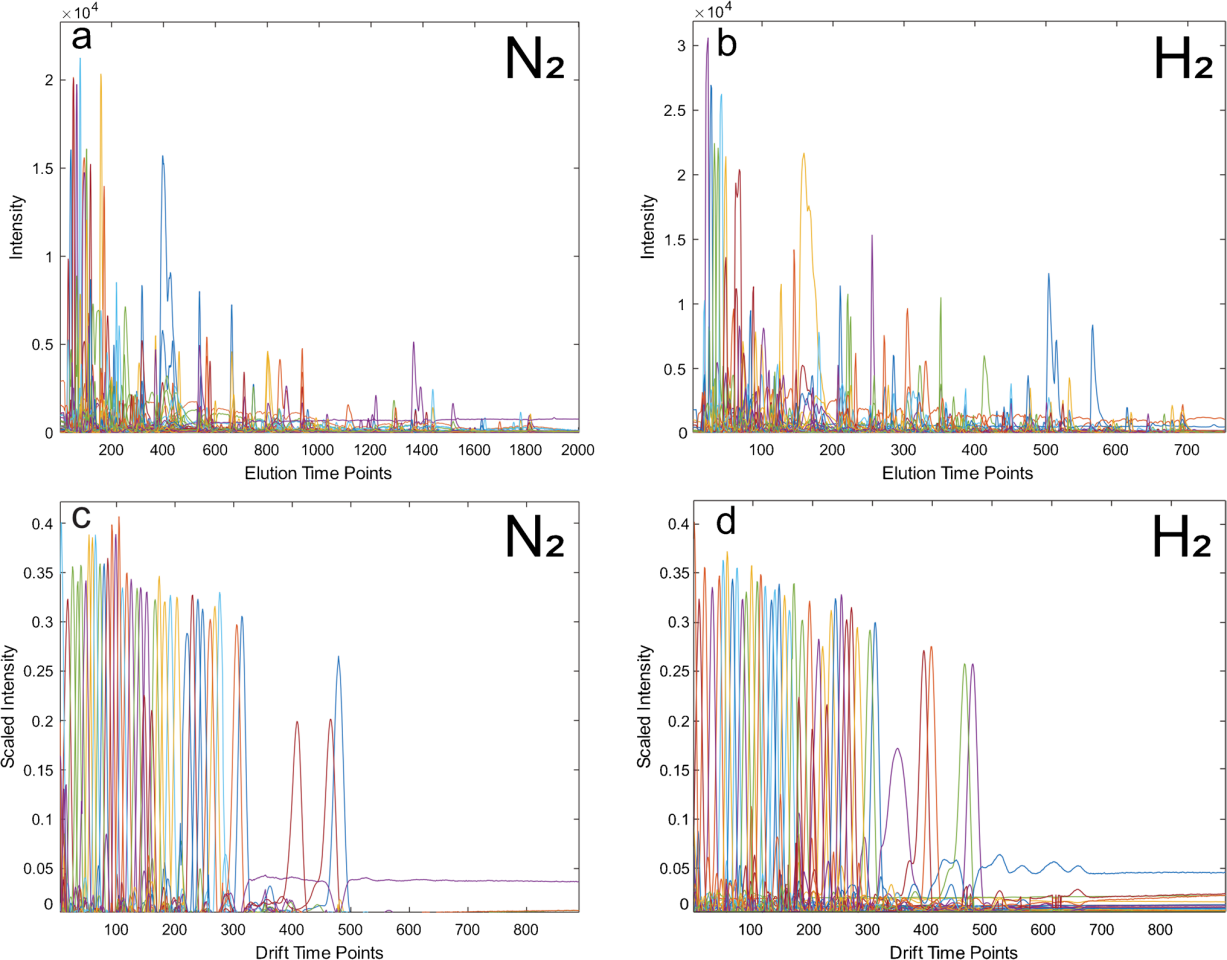
MCR-ALS resolved GC elution (**a**, **b**) and IMS drift time profiles (**c**, **d**) using the carrier gases N_2_ (left) and H_2_ (right), respectively

To further assess and confirm the improved signal-to-noise level, both datasets were subjected to MCR-ALS analysis. The resulting resolved GC elution and IMS peak profiles are visualized in Fig. 5. MCR-ALS is a powerful chemometric approach for the analysis of complex chromatographic datasets [57]. It facilitates the decomposition of multivariate data matrices into pure profiles of individual components and their corresponding concentration distributions. The resolved GC elution profiles, as well as the IMS drift time profiles obtained by MCR-ALS analysis show a similar trend and indicate that the fundamental profiles remain similar. The GC elution profiles acquired with hydrogen again exhibit a substantially higher signal intensity and an improved signal-to-noise ratio compared to those obtained with nitrogen. Since the profiles are nearly identical in direction of GC elution and IMS drift time, this suggests that the use of hydrogen as carrier gas is unlikely to introduce artifacts that would significantly affect the datasets and subsequently the data analysis. However, we would like to underline that this is only a preliminary indication and requires further investigation.

Accordingly, the MCR-ALS results also indicate an overall beneficial enhancement in signal-to-noise level when hydrogen is employed as carrier gas. As previously demonstrated in literature, MCR-ALS in combination with GC-IMS data is well suited for feature extraction [6, 58]. In this context, MCR-ALS facilitates resolving complex VOC profiles, allowing PLS-DA to capture even subtle variations in chemical compositions. In Table 3, the figures of merit of a PLS-DA model with MCR-ALS as a feature extraction step are displayed. It is important to note that first derivative and auto-scaling were used as preprocessing steps before PLS-DA. The results show a slight improvement compared to the analysis of the two datasets without MCR-ALS (see Table 2).

**Table 3:** PLS-DA Figures of Merit for the MCR-ALS calibration data, 5-fold cross-validation of the test data and the prediction of the external validation data

	N_2_		H_2_	
Calibration accuracy	1.00		0.98	
	Sensitivity	Specificity	Sensitivity	Specificity
Ghana	1.00	1.00	1.00	1.00
Ivory Coast	1.00	1.00	0.96	1.00
Nicaragua	1.00	1.00	1.00	1.00
Peru	1.00	1.00	1.00	1.00
**5-fold CV accuracy**	0.93		0.98	
	Sensitivity	Specificity	Sensitivity	Specificity
Ghana	1.00	0.94	1.00	1.00
Ivory Coast	0.85	1.00	0.96	1.00
Nicaragua	1.00	1.00	1.00	1.00
Peru	1.0	0.97	1.00	0.97
**Prediction accuracy**	0.95		0.97	
	Sensitivity	Specificity	Sensitivity	Specificity
Ghana	1.00	0.98	1.00	1.00
Ivory Coast	0.96	1.00	0.95	1.00
Nicaragua	1.00	1.00	1.00	1.00
Peru	1.00	0.98	1.00	1.00

This is in line with previous application of PLS-DA classification on MCR-ALS datasets, which yielded robust cross-validation performance, and in this study an accuracy of 0.93 for nitrogen and 0.98 for hydrogen [6]. These findings further support the analytical advantages associated with the use of hydrogen and support the need for additional investigation to clarify their implications for routine method performance.

## Conclusion

This study highlights the strong potential of non-targeted VOC profiling with hydrogen-based HS-GC-IMS for reliable cocoa mass authentication, particularly when paired with chemometrics to differentiate cocoa mass provenance. It could be demonstrated that the discrimination with PLS-DA was successful for both datasets, hydrogen and nitrogen as carrier gases, respectively, thereby highlighting the flexibility of GC-IMS. The use of hydrogen as an alternative carrier gas in HS-GC-IMS is straightforward in implementation and featured substantially shorter analysis times, along with improved data quality, more accurate and robust classification of cocoa mass samples. These technical characteristics and the use of electrolysis hydrogen offer a faster HS-GC-IMS method, which depicts a resource-friendly analytical approach in contrast to time- and energy-consuming HS-SPME-GC-MS methods. To establish volatilomics-based fingerprinting as a robust tool for cocoa authentication, it is necessary to analyze a broader range of representative samples that reflect the variability and processing conditions of cocoa products, as especially processing and fermentation conditions will have a distinct effect on the final composition of cocoa liquors and subsequently on chocolate flavor [59]. In this context, GC-IMS, as a non-targeted analytical screening technique, provides comprehensive VOC profiles of the samples and facilitates point-of-need operations, especially due to its ambient working principle and high sensitivity.

From a broader analytical perspective, this study demonstrates substantial potential for more sustainable analytical approaches in quality control of food, beverage, and flavoring products, particularly where characteristic VOC profiles serve as indicators of authenticity or quality.

## Supplementary Information

Below is the link to the electronic supplementary material.

Supplementary file1 (DOCX 95.3 KB)

## Data Availability

Weller, Philipp; Bodenbender, Lukas; Sinderhauf-Gacioch, Katrin (2026), “HS-GC-IMS data of cocoa liquor from 4 different geographies”, Mendeley Data, V1, 10.17632/684h8zcjng.1.

## References

[1] Parastar H, Weller P. How machine learning and gas chromatography-ion mobility spectrometry form an optimal team for benchtop volatilomics. Anal Chem. 2025;97:1468–81. 10.1021/acs.analchem.4c03496. 39611449 10.1021/acs.analchem.4c03496

[2] Quintanilla-Casas B, Torres-Cobos B, Bro R, Guardiola F, Vichi S, Tres A. The volatile metabolome — gas chromatography–mass spectrometry approaches in the context of food fraud. Curr Opin Food Sci. 2025;61: 101235. 10.1016/j.cofs.2024.101235.

[3] Renner G, Reuschenbach M. Critical review on data processing algorithms in non-target screening: challenges and opportunities to improve result comparability. Anal Bioanal Chem. 2023;415:4111–23. 10.1007/s00216-023-04776-7. 37380744 10.1007/s00216-023-04776-7 PMC10328864

[4] Arroyo-Manzanares N, García-Nicolás M, Castell A, Campillo N, Viñas P, López-García I, et al. Untargeted headspace gas chromatography - Ion mobility spectrometry analysis for detection of adulterated honey. Talanta. 2019;205: 120123. 10.1016/j.talanta.2019.120123. 31450393 10.1016/j.talanta.2019.120123

[5] Bordiga M, Disca V, Manfredi M, Barberis E, Carrà F, Navarini L, et al. Fingerprinting of Green Arabica Coffee Volatile Organic Compounds (VOCs): HS‐GC‐IMS Versus GC × GC‐MS. Thomas-Danguin T, editor. Int J Food Sci. 2025;2025:1302823. 10.1155/ijfo/1302823. 10.1155/ijfo/1302823 PMC12396915 40894516

[6] Parastar H, Yazdanpanah H, Weller P. Non-targeted volatilomics for the authentication of saffron by gas chromatography-ion mobility spectrometry and multivariate curve resolution. Food Chem. 2024;465:142074. 10.1016/j.foodchem.2024.142074. 39571437 10.1016/j.foodchem.2024.142074

[7] Valli E, Panni F, Casadei E, Barbieri S, Cevoli C, Bendini A, et al. An HS-GC-IMS method for the quality classification of virgin olive oils as screening support for the panel test. Foods. 2020;9: 657. 10.3390/foods9050657. 32443697 10.3390/foods9050657 PMC7278584

[8] Zacometti C, Sammarco G, Massaro A, Lefevre S, Frégière-Salomon A, Lafeuille J-L, et al. Authenticity assessment of ground black pepper by combining headspace gas-chromatography ion mobility spectrometry and machine learning. Food Res Int. 2024;179: 114023. 10.1016/j.foodres.2024.114023. 38342542 10.1016/j.foodres.2024.114023

[9] Afifah EN, Sari IA, Susilo AW, Firmanto H, Malik A, Fukusaki E, et al. Correlation between sensory attributes and metabolomic profiles of cocoa liquor from different cacao genotypes. Food Chemistry: X. 2025;28: 102498. 10.1016/j.fochx.2025.102498. 40475821 10.1016/j.fochx.2025.102498 PMC12136762

[10] Afoakwa EO. Chocolate science and technology. 2nd ed. Chichester, West Sussex, United Kingdom: John Wiley & Sons Inc; 2016. 10.1002/9781118913758.

[11] Velásquez-Reyes D, Rodríguez-Campos J, Avendaño-Arrazate C, Gschaedler A, Alcázar-Valle M, Lugo-Cervantes E. Forastero and Criollo cocoa beans, differences on the profile of volatile and non-volatile compounds in the process from fermentation to liquor. Heliyon. 2023;9: e15129. 10.1016/j.heliyon.2023.e15129. 37089295 10.1016/j.heliyon.2023.e15129 PMC10119589

[12] Braga SCGN, Oliveira LF, Hashimoto JC, Gama MR, Efraim P, Poppi RJ, et al. Study of volatile profile in cocoa nibs, cocoa liquor and chocolate on production process using GC × GC-QMS. Microchem J. 2018;141:353–61. 10.1016/j.microc.2018.05.042.

[13] Quelal-Vásconez MA, Macchioni R, Livi G, Pérez-Esteve É, Lerma-García MJ, Talens P, et al. Automatic and non-targeted analysis of the volatile profile of natural and alkalized cocoa powders using SBSE-GC-MS and chemometrics. Food Chem. 2022;389: 133074. 10.1016/j.foodchem.2022.133074. 35569247 10.1016/j.foodchem.2022.133074

[14] Rodriguez-Campos J, Escalona-Buendía HB, Orozco-Avila I, Lugo-Cervantes E, Jaramillo-Flores ME. Dynamics of volatile and non-volatile compounds in cocoa (*Theobroma cacao* L.) during fermentation and drying processes using principal components analysis. Food Res Int. 2011;44:250–8. 10.1016/j.foodres.2010.10.028.

[15] Santander Muñoz M, Rodríguez Cortina J, Vaillant FE, Escobar Parra S. An overview of the physical and biochemical transformation of cocoa seeds to beans and to chocolate: flavor formation. Crit Rev Food Sci Nutr. 2020;60:1593–613. 10.1080/10408398.2019.1581726. 30896305 10.1080/10408398.2019.1581726

[16] Liu M, Liu J, He C, Song H, Liu Y, Zhang Y, et al. Characterization and comparison of key aroma-active compounds of cocoa liquors from five different areas. Int J Food Prop. 2017;20:2396–408. 10.1080/10942912.2016.1238929.

[17] Marseglia A, Musci M, Rinaldi M, Palla G, Caligiani A. Volatile fingerprint of unroasted and roasted cocoa beans (*Theobroma cacao* L.) from different geographical origins. Food Res Int. 2020;132: 109101. 10.1016/j.foodres.2020.109101. 32331661 10.1016/j.foodres.2020.109101

[18] Bagnulo E, Scavarda C, Bortolini C, Cordero C, Bicchi C, Liberto E. Cocoa quality: chemical relationship of cocoa beans and liquors in origin identitation. Food Res Int. 2023;172: 113199. 10.1016/j.foodres.2023.113199. 37689847 10.1016/j.foodres.2023.113199

[19] Tuenter E, Delbaere C, De Winne A, Bijttebier S, Custers D, Foubert K, et al. Non-volatile and volatile composition of West African bulk and Ecuadorian fine-flavor cocoa liquor and chocolate. Food Res Int. 2020;130: 108943. 10.1016/j.foodres.2019.108943. 32156387 10.1016/j.foodres.2019.108943

[20] Poole CF, editor. Gas chromatography. 2nd ed. Amsterdam: Elsevier; 2021. 10.1016/C2010-0-66721-6

[21] Korytár P, Janssen HG, Matisová E, Brinkman UATh. Practical fast gas chromatography: methods, instrumentation and applications. TrAC Trends Anal Chem. 2002;21:558–72. 10.1016/S0165-9936(02)00811-7.

[22] Gerhardt N, Schwolow S, Rohn S, Pérez-Cacho PR, Galán-Soldevilla H, Arce L, et al. Quality assessment of olive oils based on temperature-ramped HS-GC-IMS and sensory evaluation: comparison of different processing approaches by LDA, kNN, and SVM. Food Chem. 2019;278:720–8. 10.1016/j.foodchem.2018.11.095. 30583434 10.1016/j.foodchem.2018.11.095

[23] Parastar H, Weller P. Towards greener volatilomics: is GC-IMS the new Swiss army knife of gas phase analysis? TrAC Trends Anal Chem. 2024;170: 117438. 10.1016/j.trac.2023.117438.

[24] Schanzmann H, Augustini ALRM, Sanders D, Dahlheimer M, Wigger M, Zech P-M, et al. Differentiation of monofloral honey using volatile organic compounds by HS-GCxIMS. Molecules. 2022. 10.3390/molecules27217554. 36364381 10.3390/molecules27217554 PMC9658347

[25] Nitschke A, Hitzemann M, Winkelholz J, Kirk AT, Lippmann M, Thoben C, et al. A hyper-fast gas chromatograph coupled to an ion mobility spectrometer with high repetition rate and flow-optimized ion source to resolve the short chromatographic peaks. J Chromatogr A. 2024;1736: 465376. 10.1016/j.chroma.2024.465376. 39277980 10.1016/j.chroma.2024.465376

[26] Delafield DG, Lu G, Kaminsky CJ, Li L. High-end ion mobility mass spectrometry: a current review of analytical capacity in omics applications and structural investigations. TrAC Trends Anal Chem. 2022;157: 116761. 10.1016/j.trac.2022.116761.

[27] Dodds JN, Baker ES. Ion mobility spectrometry: fundamental concepts, instrumentation, applications, and the road ahead. J Am Soc Mass Spectrom. 2019;30:2185–95. 10.1007/s13361-019-02288-2. 31493234 10.1007/s13361-019-02288-2 PMC6832852

[28] Wang S, Chen H, Sun B. Recent progress in food flavor analysis using gas chromatography-ion mobility spectrometry (GC-IMS). Food Chem. 2020;315: 126158. 10.1016/j.foodchem.2019.126158. 32014672 10.1016/j.foodchem.2019.126158

[29] Borsdorf H, Eiceman GA. Ion mobility spectrometry: principles and applications. Appl Spectrosc Rev. 2006;41:323–75. 10.1080/05704920600663469.

[30] Borsdorf H, Rudolph M. Gas-phase ion mobility studies of constitutional isomeric hydrocarbons using different ionization techniques. Int J Mass Spectrom. 2001;208:67–72. 10.1016/S1387-3806(01)00384-0.

[31] Eiceman GA, Nazarov EG, Rodriguez JE, Berglof JF. Positive reactant ion chemistry for analytical, high temperature ion mobility spectrometry (IMS): effects of electric field of the drift tube and moisture, temperature, and flow of the drift gas. Int J Ion Mobil Spectrom. 1998;1:28–37.

[32] Ewing RG, Eiceman GA, Stone JA. Proton-bound cluster ions in ion mobility spectrometry. Int J Mass Spectrom. 1999;193:57–68. 10.1016/S1387-3806(99)00141-4. 10.1016/s1387-3806(99)00141-4 11543494

[33] Brendel R, Schwolow S, Rohn S, Weller P. Comparison of PLSR, MCR-ALS and Kernel-PLSR for the quantification of allergenic fragrance compounds in complex cosmetic products based on nonlinear 2D GC-IMS data. Chemom Intell Lab Syst. 2020;205: 104128. 10.1016/j.chemolab.2020.104128.

[34] Pomareda V, Guamán AV, Mohammadnejad M, Calvo D, Pardo A, Marco S. Multivariate curve resolution of nonlinear ion mobility spectra followed by multivariate nonlinear calibration for quantitative prediction. Chemom Intell Lab Syst. 2012;118:219–29. 10.1016/j.chemolab.2012.06.002.

[35] Brendel R, Schwolow S, Rohn S, Weller P. Volatilomic profiling of *Citrus* juices by dual-detection HS-GC-MS-IMS and machine learning—an alternative authentication approach. J Agric Food Chem. 2021;69:1727–38. 10.1021/acs.jafc.0c07447. 33527826 10.1021/acs.jafc.0c07447

[36] Guckenbiehl Y, Ortner E, Rothkopf I, Buettner A. Refining and conching alter the volatile composition of dark chocolate — revealing profile changes in aroma-active volatiles and volatile organic compounds. J Agric Food Res. 2025;19:101664. 10.1016/j.jafr.2025.101664.

[37] Schmidt C, Jaros D, Rohm H. Ion mobility spectrometry as a potential tool for flavor control in chocolate manufacture. Foods Basel Switz. 2019;8. 10.3390/foods8100460. 10.3390/foods8100460 PMC6836128 31600893

[38] Bodenbender L, Rohn S, Weller P. Pushing peak shapes to perfection by high-temperature focus GC-IMS. Chemosensors. 2025;13: 131. 10.3390/chemosensors13040131.

[39] Christmann J, Rohn S, Weller P. Gc-ims-tools – a new Python package for chemometric analysis of GC–IMS data. Food Chem. 2022;394: 133476. 10.1016/j.foodchem.2022.133476. 35717914 10.1016/j.foodchem.2022.133476

[40] Lopez E, Etxebarria-Elezgarai J, Amigo JM, Seifert A. The importance of choosing a proper validation strategy in predictive models. A tutorial with real examples. Anal Chim Acta. 2023;1275: 341532. 10.1016/j.aca.2023.341532. 37524478 10.1016/j.aca.2023.341532

[41] Ballabio D, Consonni V. Classification tools in chemistry. Part 1: linear models. PLS-DA. Anal Methods. 2013;5: 3790. 10.1039/c3ay40582f.

[42] Montesinos López OA, Montesinos López A, Crossa J. Overfitting, Model Tuning, and Evaluation of Prediction Performance. Multivar Stat Mach Learn Methods Genomic Predict. Cham: Springer International Publishing; 2022. pp. 109–39. 10.1007/978-3-030-89010-0_4.

[43] Wong T-T. Performance evaluation of classification algorithms by k-fold and leave-one-out cross validation. Pattern Recognit. 2015;48:2839–46. 10.1016/j.patcog.2015.03.009.

[44] Quelal OM, Hurtado DP, Benavides AA, Alanes PV, Alanes NV. Key aromatic volatile compounds from roasted cocoa beans, cocoa liquor, and chocolate. Fermentation. 2023;9: 166. 10.3390/fermentation9020166.

[45] Garcia‐Esteban M, Ansorena D, Astiasaran I, Martin D, Ruiz J. Comparison of simultaneous distillation extraction (SDE) and solid‐phase microextraction (SPME) for the analysis of volatile compounds in dry‐cured ham. J Sci Food Agric. 2004;84:1364–70. 10.1002/jsfa.1826.

[46] Jordán MJ, Goodner KL, Shaw PE. Characterization of the aromatic profile in aqueous essence and fruit juice of yellow passion fruit (*Passiflora edulis* Sims F . flavicarpa degner ) by GC−MS and GC/O. J Agric Food Chem. 2002;50:1523–8. 10.1021/jf011077p. 11879031 10.1021/jf011077p

[47] Fadel HHM, Abdel Mageed MA, Abdel Samad AKME, Lotfy SN. Cocoa substitute: evaluation of sensory qualities and flavour stability. Eur Food Res Technol. 2006;223:125–31. 10.1007/s00217-005-0162-3.

[48] Menezes AGT, Batista NN, Ramos CL, Silva ARDAE, Efraim P, Pinheiro ACM, et al. Investigation of chocolate produced from four different Brazilian varieties of cocoa (*Theobroma cacao* L.) inoculated with *Saccharomyces cerevisiae*. Food Res Int. 2016;81:83–90. 10.1016/j.foodres.2015.12.036.

[49] Ullrich L, Casty B, André A, Hühn T, Steinhaus M, Chetschik I. Decoding the fine flavor properties of dark chocolates. J Agric Food Chem. 2022;70:13730–40. 10.1021/acs.jafc.2c04166. 36255101 10.1021/acs.jafc.2c04166

[50] Siegmund B, Murkovic M. Changes in chemical composition of pumpkin seeds during the roasting process for production of pumpkin seed oil (part 2: volatile compounds). Food Chem. 2004;84:367–74. 10.1016/S0308-8146(03)00241-3.

[51] Quijano CE, Salamanca G, Pino JA. Aroma volatile constituents of Colombian varieties of mango (*Mangifera indica *L.). Flavour Fragr J. 2007;22:401–6. 10.1002/ffj.1812.

[52] Tretyakov, KV. Retention Data. NIST Mass Spectrometry Data Center; 2007. https://webbook.nist.gov/cgi/cbook.cgi?Author=Tret%27yakov%2C+K.&Units=SI&Mask=2000.

[53] Gromski PS, Muhamadali H, Ellis DI, Xu Y, Correa E, Turner ML, et al. A tutorial review: metabolomics and partial least squares-discriminant analysis – a marriage of convenience or a shotgun wedding. Anal Chim Acta. 2015;879:10–23. 10.1016/j.aca.2015.02.012. 26002472 10.1016/j.aca.2015.02.012

[54] Song W, Wang H, Maguire P, Nibouche O. Collaborative representation based classifier with partial least squares regression for the classification of spectral data. Chemom Intell Lab Syst. 2018;182:79–86. 10.1016/j.chemolab.2018.08.011.

[55] Christmann J, Rohn S, Weller P. Finding features - variable extraction strategies for dimensionality reduction and marker compounds identification in GC-IMS data. Food Res Int. 2022;161: 111779. 10.1016/j.foodres.2022.111779. 36192933 10.1016/j.foodres.2022.111779

[56] Farrés M, Platikanov S, Tsakovski S, Tauler R. Comparison of the variable importance in projection (VIP) and of the selectivity ratio (SR) methods for variable selection and interpretation. J Chemom. 2015;29:528–36. 10.1002/cem.2736.

[57] De Juan A, Tauler R. Multivariate curve resolution: 50 years addressing the mixture analysis problem – a review. Anal Chim Acta. 2021;1145:59–78. 10.1016/j.aca.2020.10.051. 33453882 10.1016/j.aca.2020.10.051

[58] Parastar H, Weller P. Feature selection and extraction strategies for non-targeted analysis using GC-MS and GC-IMS: a tutorial. Anal Chim Acta. 2025;1343: 343635. 10.1016/j.aca.2025.343635. 39947788 10.1016/j.aca.2025.343635

[59] Gopaulchan D, Moore C, Ali N, Sukha D, Florez González SL, Herrera Rocha FE, et al. A defined microbial community reproduces attributes of fine flavour chocolate fermentation. Nat Microbiol. 2025;10:2130–52. 10.1038/s41564-025-02077-6. 40825855 10.1038/s41564-025-02077-6 PMC12408344

