# Supplementary material for: Towards “greener” strategies in quality control: rapid volatilomics of cocoa based on HS-GC-IMS and machine learning
Source: Anal Bioanal Chem. 2026 Mar 10;418(16):5205–17. doi: 10.1007/s00216-026-06415-3 (PMC13423914; doi:10.1007/s00216-026-06415-3)
Supplement: Supplementary file 1 — Supplementary file1 (DOCX 95.3 KB) [file 216_2026_6415_MOESM1_ESM.docx]

Electronic supplementary material

# Towards “greener” strategies in quality control: rapid volatilomics of cocoa based on HS-GC-IMS and machine learning

Lukas Bodenbender^1,2^, Sascha Rohn^2^, Hadi Parastar^3^, Katrin Sinderhauf-Gacioch^4^, Philipp Weller^1*^

^1^Institute for Instrumental Analysis and Bioanalytics, Technische Hochschule Mannheim, Paul-Wittsack-Str. 10, 68163 Mannheim, Germany

^2^Department of Food Chemistry and Analysis, Institute of Food, Technology and Food Chemistry, Technische Universität Berlin, KAA 1-2, Kaiserin-Augusta-Allee 14, 10553 Berlin, Germany

^3^Department of Chemistry, Sharif University of Technology, P.O. Box 11155-9516, Tehran, Iran

^4^ Alfred Ritter GmbH & Co. KG, Alfred-Ritter-Strasse 25, 7111 Waldenbuch, Germany

*corresponding author, *E-mail address:* p.weller@hs-mannheim.de (P. Weller)


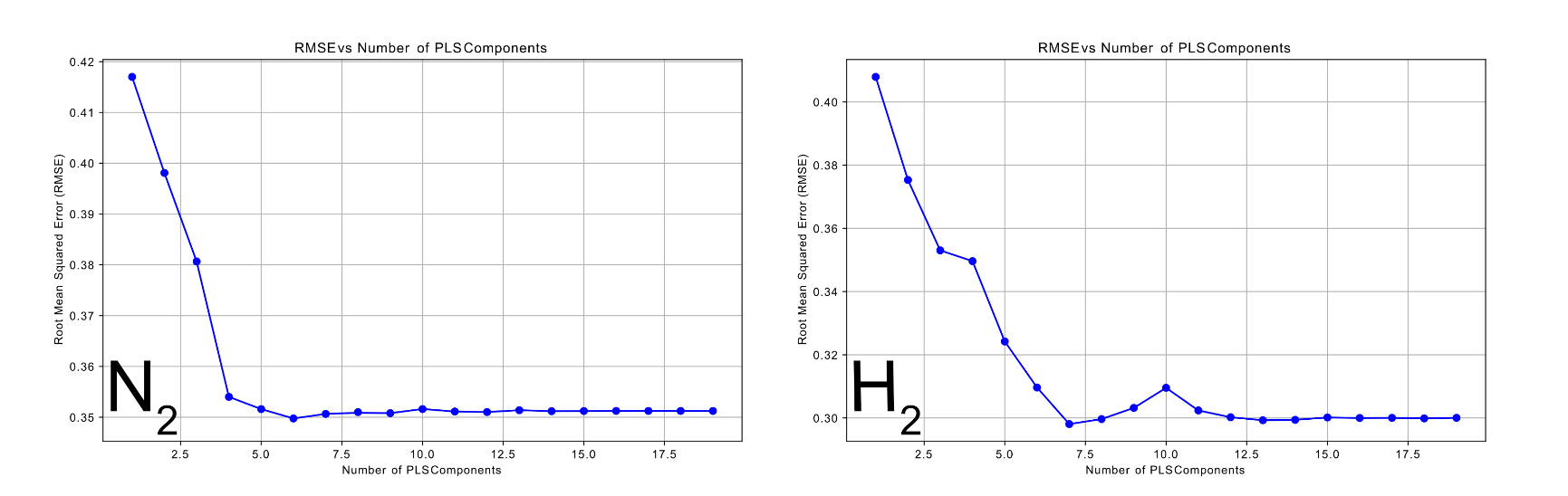


Figure S1: RMSE plot versus number of latent variables (LVs) for PLS model for the carrier gas nitrogen (left) and hydrogen (right), respectively.
